# Health care and contraceptive decision-making autonomy and use of female sterilisation among married women in Malawi

**DOI:** 10.3389/fgwh.2024.1264190

**Published:** 2024-06-04

**Authors:** Nurudeen Alhassan

**Affiliations:** Population Dynamics and Demographic Dividend Thematic Area, African Institute for Development Policy (AFIDEP), Lilongwe, Malawi

**Keywords:** decision-making autonomy, health care, contraceptive, female sterilisation, Malawi

## Abstract

**Introduction:**

Female sterilisation is the most common contraceptive method used globally. The use of female sterilisation is disproportionately low in sub-Sahara Africa (SSA) at just 1%. Nonetheless, the prevalence of sterilisation among married women in Malawi is quite high at about 11%. While a few recent studies in SSA have examined the relationship between women's decision-making autonomy and use of long-acting contraceptives, very few have investigated whether different dimensions of decision-making autonomy predict the use of female sterilisation differently. The objective of this study was therefore to examine the relationship between health care and contraceptive decision-making autonomy and the use of female sterilisation in Malawi.

**Data and methods:**

The study relied on secondary data from the 2015–16 Malawi Demographic and Health Survey. The sample comprised 9,164 married women in Malawi that were using a modern contraceptive. Multinomial logistic regression analysis was used to examine the association between health care and contraceptive decision-making autonomy and the use of female sterilisation, controlling for key socio-demographic characteristics.

**Results:**

The study revealed that the percentage of married women that made health care and contraceptive decisions independently was quite low. The main finding of this study was that contraceptive decision-making autonomy increased the relative likelihood of using female sterilisation while health care autonomy was associated with a lower likelihood of being sterilized. The socio- demographic characteristics that significantly predicted the use of female sterilisation included age, place of residence, household wealth and the number of children a woman had.

**Conclusion:**

This study demonstrates that health care and contraceptive decision making have different effects on the use of female sterilisation among married women in Malawi. Specifically, women with autonomy in health care decision making had a relatively lower likelihood of using female sterilisation while those with contraceptive decision-making autonomy had a higher likelihood of using female sterilisation. This suggests that intervention aimed at increasing the uptake of female sterilisation in Malawi need to focus on empowering women in the contraceptive decision-making domain.

## Introduction

Contraceptive use helps individual women and couples to achieve their reproductive goals and exercise the right to have children by choice (1, 2). Modern contraceptive use has extensive benefits including preventing unintended pregnancies and their associated consequences such as maternal and child mortality (3–5). Long-term contraceptives which include both long-acting reversible contraceptives (LARCs) and permanent methods such female sterilisation and vasectomy have been cited as the most effective contraceptive methods available to women, men and couples (6). Female sterilisation is an irreversible contraceptive method that involves surgical procedures, with the most common procedures being postpartum tubal ligation, laparoscopic tubal disruption or salpingectomy, and hysteroscopic tubal occlusion (7). Globally, nearly a quarter (24%) of all women of reproductive age using any contraceptive method(s) rely on female sterilisation. There are huge regional variations in the use of female sterilisation, with prevalence highest in Central and Southern Asia (21.8%) followed by Latin America and the Caribbean (16%) (8). Sub-Saharan Africa (SSA) has the lowest prevalence of female sterilisation in the world, estimated at just 1.1% (8). Yet, there is evidence that demand for family planning to limit childbearing is increasing in the region (9).

The main barriers to the use of long-acting contraceptives including female sterilisation in SSA include limited provider expertise, lack of awareness, fear of side effects and poverty (10–12). Notwithstanding these barriers and the generally low prevalence of female sterilisation across countries in SSA, Malawi has experienced one of the most remarkable increases in the uptake of female sterilisation in the sub- region (13, 14). It is important to note that the most common contraceptive methods used by all women of reproductive age in Malawi are injectables (22.5%), implants (9.0%) and female sterilisation (8.3%) (13). The prevalence of female sterilisation among married women in Malawi increased from 1.7% in 1992 to approximately 11% in 2016 (13, 15). The remarkable increase in the use of female sterilisation has occurred in both rural and urban areas, with prevalence among married women in both settings being equal (13). The outstanding progress in the uptake of female sterilisation in Malawi has been attributed to policy changes and programmatic interventions (14). Mobile and outreach clinics run by private not-for-profit organisations such as Banja La Mtsogolo (the Malawian affiliate of Marie Stopes International) have been key in extending access to female sterilisation in rural and remote communities (10).

The rapid increase in the use of female sterilisation in Malawi, however, raises questions about the potential of service providers and other individuals including sexual partners to undermine women's reproductive autonomy. Given the history of forced sterilisation targeted at marginalized and poor women (16, 17), it is imperative to establish that the increasing uptake of female sterilisation in Malawi is the free choice of women (18). Even though a number of recent studies in SSA have examined the relationship between women's decision-making autonomy and use of long-acting contraceptives, the findings so far have been contradictory and inconclusive (12, 19–22). For instance, Adde et al., in a study of eleven SSA countries found that women with higher decision-making autonomy had a greater likelihood of using long-acting contraceptives (19). Bolarinwa et al., on the other hand, in a study of twenty SSA countries found women who were not involved in household decision-making were more likely to use long-acting methods (12). With specific reference to female sterilisation, one study conducted in Uganda found that the odds being sterilized were higher for women who reported their husband/partner as the main decision-maker for contraceptive use and those who made such decisions jointly with their husbands/partners compared to women that made contraceptive decisions alone (21).

In addition to the contradictory findings, no study to the best of my knowledge in SSA has examined whether different dimensions of women's decision-making autonomy predict the use of female sterilisation differently. For a country that has one of the highest prevalence rates of female sterilisation in SSA, the relationship between women's decision-making autonomy and the use of female sterilisation still remains unknown in Malawi. The objective of this study was therefore to examine whether health care and contraceptive decision-making autonomy predict the use of female sterilisation differently among married women in Malawi.

## Conceptual framework

**Figure 1:**
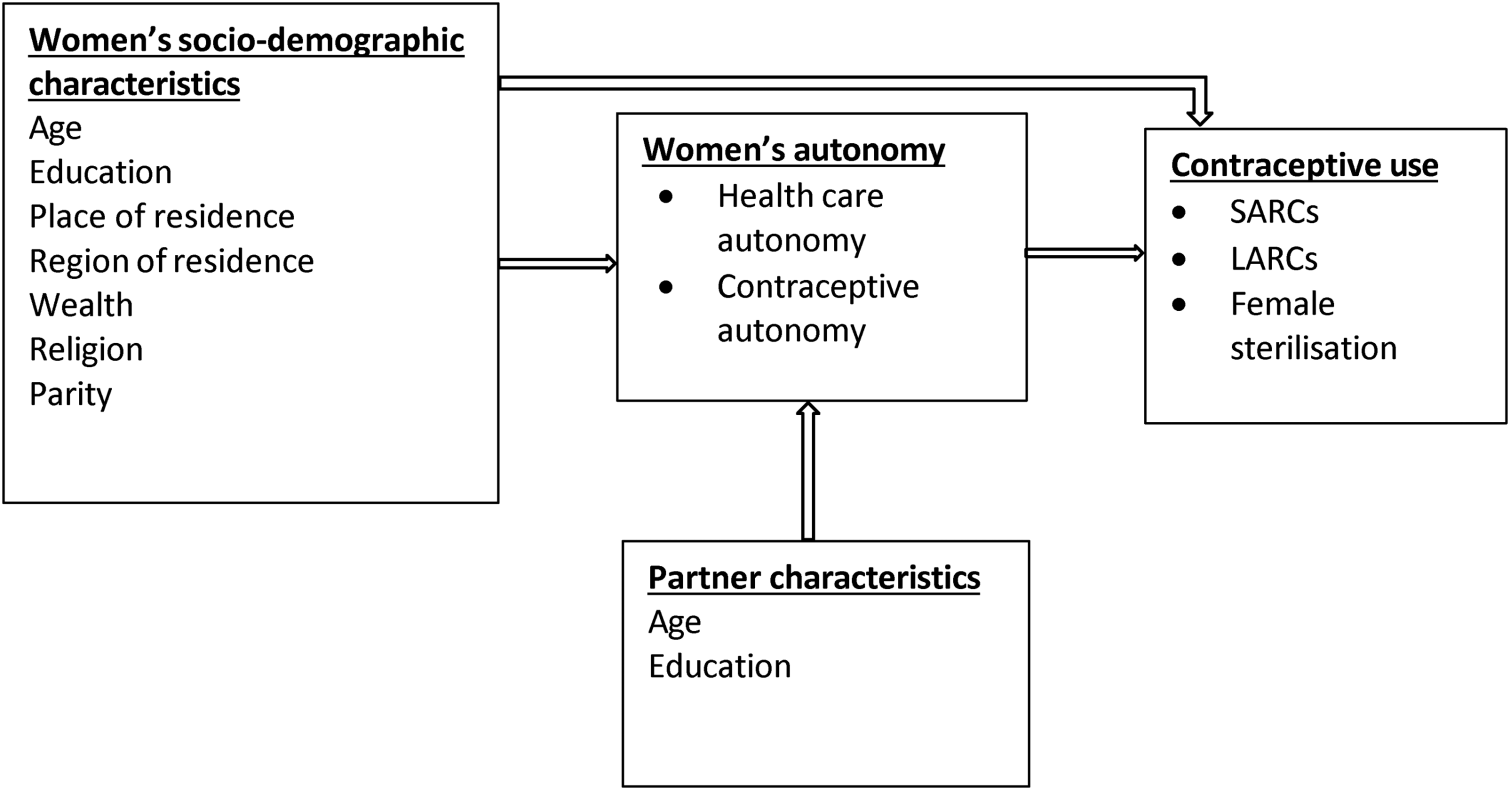
Conceptual framework.

Figure 1 presents the conceptual framework that guided the current study. The conceptual framework was informed by existing literature on women's decision-making autonomy and reproductive behavior (19, 23–27). Decision-making autonomy is broadly defined as the freedom and independence of women to make their own choices and decisions (24, 28). Decision-making autonomy is a multifaceted concept with several dimensions including financial, health care, contraceptive and movement autonomy. For this study, the focus is on two dimensions of decision-making autonomy: health care and contraceptive autonomy. Health care autonomy is the ability of women to make decisions about their health and to utilize health care services while contraceptive autonomy is the freedom and independence of women to make decisions specifically about contraceptive use (24). There is evidence that women's decision-making autonomy is influenced and constrained by demographic, socioeconomic and cultural factors such as women's age, education, wealth and religion as well as the characteristics of their partners (24, 29). There is also sufficient evidence that women's decision-making autonomy increases contraceptive use (27).

Even though both health care and contraceptive decision-making autonomy may increase the use of contraceptives, I argue that these two domains of decision-making autonomy are different when it comes to their effect on specific types of contraceptives. Specifically, I hypothesize that contraceptive decision-making autonomy predicts a greater likelihood of using female sterilisation compared to autonomy in health care decision-making. The underlying assumption of this hypothesis is that women with autonomy in contraceptive decision-making have greater control and are able overcome the relatively higher sociocultural constraints to using an irreversible/permanent contraceptive method than women with autonomy in the health care domain. Contraceptive use in SSA has mainly been driven by the desire to space and not to limit births (30). Fertility limitation in marriage including the use of sterilisation to achieve that aim has traditionally been discouraged. Therefore, the cultural and normative constraints to using permanents methods such as female sterilisation are much higher than those for using LARCs and short-acting reversible contraceptives (SARCs). Overcoming these constraints thus require greater autonomy in contraceptive decision-making than in health care.

## Methods

### Data and study design

The current study analysed data from the 2015–16 Malawi Demographic and Health Survey, which is the latest DHS survey in the country. The survey used a two-stage stratified sampling technique to select a total of 25,146 eligible women for the survey. Out of the 25,146 eligible women, a total of 24,564 women were actually interviewed. A detailed description of the sampling procedure of the survey is available in the final report (13). The data for this paper is from a sub-sample of 9,164 women in union (married/living with a partner) that were currently using a modern contraceptive(s). The data for this analysis was weighted to account for unequal sampling probabilities, and also to consider the complexity (clustering and stratification) of the DHS sampling design.

### Study variables

The outcome variable of this study “current use of any modern contraceptive(s)” was categorized into three: (a) short-acting reversible contraceptives (SARCs)-injectables, pills, condoms, emergency contraceptives etc.) (b) long-acting reversible contraceptives (LARCs)─implants and intrauterine devices(IUDs), and (c) female sterilisation. The two main predictor variables were: (a) women's autonomy over health care decision-making and (b) women's autonomy over decision-making for contraceptive use. The first of these two variables “health care autonomy” was generated from the DHS question that asked women about the “person who usually decides on respondent's health care”. This question had five response options: (a) respondent alone (b) respondent and husband/partner (c) husband/partner alone (d) someone else and (e) other. I recoded these response option into three by dropping the last two response options, “someone else” and “other”, which had very few cases while maintaining all the other response options. Thus, the final health care autonomy variable was categorized as: (a) the woman (b) Joint (woman and husband/partner) (c) husband/partner alone. In this study, a woman was considered to have autonomy over healthcare decision-making if she alone made decisions regarding her health; a woman had some autonomy if decision-making was joint (the woman and her husband/partner); while she had no autonomy if decision-making on her health were made by the husband/partner alone.

The other predictor variable “contraceptive autonomy” was measured using the question in the survey that asked women about the “decision maker for using contraception”. The response options were: (a) mainly respondent (b) mainly husband/partner (c) Joint decision and (d) other. There were very few cases in the “other” response category, so these cases had to be dropped from the analysis. The final “contraceptive autonomy” variable therefore had three categories: (a) mainly respondent (b) mainly husband/partner and (c) joint decision. Like the health care autonomy variable, a woman was considered to have contraceptive autonomy if she was the main decision maker for contraceptive use. A woman was deemed to have some contraceptive autonomy if decisions for contraceptive use were made jointly with her husband/partner while a woman had no contraceptive autonomy if decisions for contraceptive use were mainly made by her husband/partner.

I controlled for the effect of other key variables, in the multivariate analysis, that had the potential to confound the relationship between women's decision-making autonomy and the use of modern contraceptives. The control variables included were key socio-demographic characteristics of the women and their partners. See Table 1 for the list of control variables and their measurement in this study.

**Table 1:** Characteristics of study sample and associations with healthcare and contraceptive autonomy.

Socio-demographic	All women	Health care autonomy, *n* (%)				Contraceptive autonomy, *n* (%)			
	*n* (%)	Woman	Joint^a^	Partner	*P*-value	Woman	Joint	Partner	*P*-value
Characteristics	9,164 (100%)	1,681 (18.1%)	4,803 (51.8%)	2,629 (30.1%)		1,173 (12.9%)	7,335 (80.6%)	656 (6.5%)	
Age									
15–24	2,422 (26.5%)	391 (15.5%)	1,249 (51.2%)	762 (33.3%)	0.0062	219 (9.2%)	2,005 (83.3%)	198 (7.5%)	<0.001
25–39	5,299 (57.6%)	984 (18.8%)	2,802 (51.9%)	1,490 (29.3%)		686 (13.2%)	4,253 (80.5)	360 (6.3%)	
40+	1,443 (15.9%)	306 (19.7%)	752 (52.6%)	377 (27.7%)		268 (18.2%)	1,077 (76.1%)	98 (5.7%)	
Education									
No education	1,095 (13.0%)	189 (17.4%)	567 (51.1%)	334 (31.5%)	<0.001	172 (17.1%)	811 (74.6%)	112 (8.3%)	<0.001
Primary	5,953 (65.3%)	1,088 (17.8%)	2,972 (49.7%)	1,863 (32.5%)		769 (12.7%)	4,755 (80.5%)	429 (6.8%)	
Secondary/higher	2,116 (21.6%)	404 (19.4%)	1,264 (58.6%)	432 (22.0%)		232 (11.1%)	1,769 (84.4%)	115 (4.5%)	
Place of residence									
Rural	7,341 (82.9%)	1,325 (17.5%)	3,731 (50.6%)	2,244 (31.9%)	<0.001	910 (12.2%)	5,849 (80.5%)	582 (7.4%)	<0.001
Urban	1,823 (17.1%)	356 (21.2%)	1,072 (57.8%)	385 (21.1%)		263 (16.6%)	1,486 (81.1%)	74 (2.2%)	
Region of residence									
Central	3,389 (46.9%)	557 (17.9%)	1,787 (51.2%)	1,034 (30.9%)	0.0056	345 (10.4%)	2,876 (85.0%)	168 (4.5%)	<0.001
Sothern	4,016 (41.6%)	698 (17.0%)	2,166 (54.1%)	1,122 (28.9%)		578 (15.8%)	3,094 (75.9%)	344 (8.3%)	
Northern	1,759 (11.5%)	426 (22.9%)	850 (46.2%)	473 (30.9%)		250 (12.5%)	1,365 (79.2%)	144 (8.3%)	
Wealth status									
Poorest	1,649 (18.0%)	342 (21.2%)	787 (45.8%)	509 (33.0%)	<0.001	248 (14.6%)	1,280 (78.5%)	121 (6.9%)	0.0258
Poorer	1,816 (21.1%)	304 (16.5%)	915 (50.8%)	586 (32.7%)		237 (13.1%)	1,437 (79.6%)	142 (7.2%)	
Middle	1,844 (21.0%)	330 (17.8%)	975 (52.0%)	531 (30.2%)		230 (12.4%)	1,473 (81.2%)	141 (6.4%)	
Richer	1,881 (20.3%)	345 (16.9%)	1,020 (53.6%)	508 (29.6%)		250 (14.4%)	1,502 (79.7%)	129 (5.9%)	
Richest	1,974 (19.7%)	360 (18.5%)	1,106 (56.4%)	495 (25.1%)		208 (10.1%)	1,643 (83.7%)	123 (6.2%)	
Religious affiliation									
Other religion	4,125 (45.1%)	743 (17.8%)	2,126 (51.0%)	1,229 (31.2%)	0.0009	520 (12.1%)	3,284 (80.9%)	321 (6.9%)	<0.001
CCAP	1,465 (18.0%)	261 (18.9%)	803 (53.4%)	391 (27.7%)		153 (9.8%)	1,212 (84.0%)	100 (6.2%)	
Catholic	1,616 (18.3%)	285 (16.2%)	885 (54.3%)	440 (29.5%)		177 (11.7%)	1,324 (82.2%)	115 (6.1%)	
SDA/Baptist	664 (6.8%)	125 (17.6%)	393 (59.5%)	145 (22.9%)		90 (13.7%)	533 (80.7%)	41 (5.6%)	
Anglican	422 (2.4%)	79 (17.5%)	208 (49.9%)	134 (32.6%)		60 (20.9%)	343 (72.1%)	19 (7.0%)	
Muslim	816 (9.3%)	172 (20.9%)	360 (43.3%)	278 (35.8%)		164 (21.6%)	600 (72%)	52 (6.4%)	
Number of children									
Mean (S.D)	3.4	3.5	3.3	3.4	0.0003	3.7	3.3	3.4	<0.001
Partner age in years									
15–24	912 (10.4%)	152 (16.2%)	445 (48.6%)	309 (35.2%)	0.0021	76 (8.7%)	753 (84.0%)	83 (7.3%)	<0.001
25–39	5,172 (56.3%)	895 (17.3%)	2,734 (51.8%)	1,510 (30.9%)		586 (11.8%)	4,235 (81.7%)	351 (6.5%)	
40–49	2,213 (23.9%)	444 (19.9%)	1,180 (52.6%)	581 (27.5%)		351 (15.1%)	1,705 (78.7%)	157 (6.3%)	
50+	867 (9.4%)	190 (20.6%)	444 (53.5%)	229 (25.9%)		160 (19.1%)	642 (74.6%)	65 (6.3%)	
Partner education									
No education	854 (9.7%)	146 (16.9%)	439 (50.6%)	265 (32.5%)	<0.001	154 (18.3%)	619 (74.0%)	81 (7.7%)	<0.001
Primary	4,850 (54.6%)	866 (17.3%)	2,431 (49.8%)	1,529 (32.9%)		624 (12.9%)	3,869 (80.2%)	357 (6.9%)	
Secondary	2,917 (30.2%)	569 (19.1%)	1,585 (54.0%)	743 (26.9%)		338 (11.7%)	2,385 (82.4%)	194 (5.9%)	
High	543 (5.4%)	100 (22.2%)	348 (62.2%)	92 (15.6%)		57 (10.8%)	462 (85.5%)	24 (3.7%)	

^a^ Decisions about health care or contraceptive use made by women and their partners.

Malawi Demographic and Health Survey, 2015–16.

### Statistical analysis

Descriptive statistics; frequencies, mean and cross-tabulations were generated to show the pattern of distribution of key variables as well as the association between key socio-demographic characteristics and the two autonomy variables. Pearson's chi-square test was conducted to test for statistically significant associations between key socio-demographic characteristics and women's decision-making autonomy. Multinomial logistic regression analysis was then used to examine the association between health care and contraceptive decision-making autonomy and the use of female sterilisation. Before fitting the regression models, we conducted correlation analysis which established that the two autonomy variables were independent of each other. In all, we fitted two multinomial logistic regression models. In stage one (unadjusted model), a logistic regression model was fitted to examine the relationship between the control variables and the outcome. In stage two (adjusted model), I examined the relationship between contraceptive and health care decision-making autonomy and the use of female sterilisation when controlling for the sociodemographic characteristics of the women and their husbands/partners as in the unadjusted model. The reference group in the regression analysis was the users of LARCs (implants and IUDs). All the analyses were conducted using STATA 17, and the logistic regression results were presented as relative risk ratios (RRR) with 95% confidence intervals and *p* values (*p* < 0.05 was considered significant).

## Results

### Characteristics of study sample and associations with decision-making autonomy

The results of this study showed that about six in ten married women in Malawi using a modern contraceptive(s) relied on SARCs. Approximately 22% of the sample used LARCs while the remaining 18% of them had undergone sterilisation.

Table 1 shows the distribution of the study sample by key socio-demographic characteristics and the associations between these characteristics and the two decision-making autonomy variables, healthcare and contraceptive autonomy. Overall, 18% of the women had autonomy to make decisions about their own health while approximately 13% had autonomy to make decisions about contraceptive use. A slight majority (52%) of the women made decisions about their health jointly with their husbands/partners while overwhelming majority (81%) of them made contraceptive use decisions jointly with their husbands/partners. For approximately 7% of women, decisions about contraceptive use was made by their husbands/partners alone compared to about a third (30%) of women whose husbands/partners alone made decisions about health care for them.

Majority (58%) of the women in this study were aged 25–39 years while about 16% of them were 40 years and above, with a little over a quarter (27%) being youth aged 15–24 years. Age was positively associated with both health care and contraceptive decision-making autonomy. A higher percentage of the women aged 40+ years had autonomy to make decisions about health care and contraceptive use. With regards to education, almost two-thirds (65%) of the sample had primary education while about two in ten of them had secondary or higher education. Education was significantly associated with both health care and contraceptive autonomy. A slightly higher percentage (19.4%) of the women with secondary or higher education had health care autonomy compared to those with no education (17.1%) and primary education (17.8%). However, we found that the percentage of women with no education and primary education (17.1% and 12.7%) that had contraceptive autonomy was higher than those with secondary or higher education (11.1%).

As expected, an overwhelming majority of the women were resident in rural areas (82.9%). Residence in an urban setting was positively associated with both health care and contraceptive autonomy. Contrary to expectation, household wealth status was negatively associated with both health care and contraceptive autonomy. Overall, a higher percentage of women in the poorest households had health care and contraceptive use autonomy compared to those in poorer, middle, richer and richest households. Close to half (45%) of the women in this study were Christians affiliated to churches other than the main Christian denominations in Malawi. The percentage (18.0%) of women affiliated to the Church of Central Africa Presbyterian (CCAP) was similar to the percentage affiliated to the Catholic Church (18.3%). About 9% of the sample was comprised of women affiliated to Islam while 2.4% of them were Anglicans. Religion was significantly associated with both health care and contraceptive decision-making autonomy. In terms of parity, we found a significant association between the average number of children a woman had and both health care and contraceptive autonomy. On average, the women with health care and contraceptive autonomy had a slightly higher number of children than those who made healthcare and contraceptive decisions jointly with partners and those whose partners made such decisions for them.

As mentioned previously, we included two characteristics of the women's partners/husbands in the analysis: age and education. The results show that the age of partners was significantly associated with health care and contraceptive autonomy. The age of partners was positively associated with health care and contraceptive autonomy. A higher percentage of women with partners aged 50 + years had health care and contraceptive autonomy compared to those with partners younger than 50 years. The educational attainment of partners was significantly associated with both health care and contraceptive autonomy. Partner education was positively associated with health care autonomy but negatively associated with contraceptive autonomy. A higher percentage (22.2%) of women with partners of higher education had health care autonomy compared to those with partners of no education and primary education (16.9% and 17.3% respectively). However, a higher percentage of women with partners of no education (18.3%) had contraceptive autonomy compared to those with partners of secondary (11.7%) and higher education (10.8%).

### Effect of women's autonomy and socio-demographic characteristics on use of female sterilisation among married women in Malawi

Table 2 presents the results of the multinomial logistic regression analysis (unadjusted model) showing the effect of the socio-demographic characteristics on the use of female sterilisation. As indicated earlier, we run two multinomial logistic regression models. The unadjusted model (Table 2) examined the effect of the socio-demographic characteristics on the use of female sterilisation while the adjusted model (Table 3) examined the effect of women's health care and contraceptive decision- making autonomy on the outcome when adjusting for socio-demographic characteristics.

**Table 2:** Multinomial logistic regression analyses (unadjusted model) showing the effect of socio- demographic characteristics on use of female sterilisation and short-acting contraceptives relative to LARCs.

Variables included in analysis	Female sterilisation vs. LARCs		SARCs vs. LARCs	
	RRR	95% CI	RRR	95% CI
Age				
25–39 (ref.)	1.00	–	1.00	–
15–24	0.04*	0.01–0.11	1.22	0.97–1.54
40+	3.54*	2.61–4.80	1.67*	1.25–2.23
Education				
No education	1.00	–	1.00	–
Primary	0.86	0.65–1.14	0.92	0.74–1.14
Secondary/higher	0.45*	0.30–0.66	0.84	0.65–1.09
Place of residence				
Rural	1.00	–	1.00	–
Urban	1.47*	1.03–2.10	1.00	0.77–1.30
Region of residence				
Central	1.00	–	1.00	–
Southern	0.55*	0.43–0.70	1.25*	1.04–1.51
Northern	0.43*	0.30–0.61	0.81	0.64–1.03
Wealth status				
Poorest	1.00	–	1.00	–
Poorer	1.17	0.84–1.63	0.98	0.79–1.21
Middle	1.27	0.90–1.80	0.97	0.78–1.21
Richer	1.36	0.99–1.86	0.89	0.72–1.11
Richest	1.54*	1.10–2.16	0.80	0.62–1.01
Religious affiliation				
Other religion	1.00	–	1.00	
CCAP	1.30	0.98–1.43	1.18	0.97–1.73
Catholic	1.32*	1.00– 1.45	1.21*	1.00–1.74
SDA/Baptist	1.27	0.95–1.63	1.24	0.83–1.95
Anglican	0.73	0.48–1.05	0.71	0.43–1.24
Muslim	0.73	1.46–2.64	1.96*	0.48–1.11
Number of living children				
Mean (S.D)	1.12*	1.04–1.21	0.91*	0.86–0.97
Partner age in years				
15–24	1.00	–	1.00	–
25–39	2.53	0.70–9.14	0.91	0.72–1.15
40–49	8.5*	2.30–31.41	1.19	0.88–1.60
50+	12.3*	3.19– 47.17	0.95	0.62–1.47
Partner education				
No education	1.00	–	1.00	–
Primary	1.21	0.87–1.67	1.19	0.95–1.49
Secondary	0.74	0.51–1.09	0.95	0.72–1.24
High	0.74	0.38–1.41	0.55*	0.36–0.85

* *p* < 0.05; RRR, Relative Risk Ratio.

**Table 3:** Multinomial logistic regression analyses (adjusted model) showing the effect of decision- making autonomy and socio-demographic characteristics on use of female sterilisation and short- acting contraceptives relative to LARCs.

Variables included in analysis	Female sterilisation vs. LARCs		SARCs vs. LARCs	
	RRR	95% CI	RRR	95% CI
Healthcare autonomy				
Joint (ref.)	1.00	–	1.00	–
Woman	0.60*	0.45–0.80	1.12	0.92–1.36
Partner	0.89	0.70–1.13	1.06	0.91–1.25
Contraceptive autonomy				
Joint (ref.)	1.00	–	1.00	–
Woman	1.40*	1.05–1.86	1.26*	1.02–1.55
Partner	0.76	0.51–1.13	1.02	0.80–1.31
Age				
25–39 (ref.)	1.00	–	1.00	–
15–24	0.04*	0.01–0.11	1.22	0.97–1.53
40+	3.55*	2.62–4.83	1.67*	1.25–2.23
Education				
No education	1.00	–	1.00	–
Primary	0.87	0.65–1.16	0.91	0.74–1.13
Secondary/higher	0.46*	0.31–0.68	0.83	0.64–1.08
Place of residence				
Rural	1.00	–	1.00	–
Urban	1.42*	1.01–2.02	0.99	0.77–1.29
Region of residence				
Central	1.00	–	1.00	–
Southern	0.54*	0.42–0.69	1.26*	1.05–1.51
Northern	0.43*	0.30–0.61	0.80	0.63–1.01
Wealth status				
Poorest	1.00	–	1.00	
Poorer	1.14	0.81–1.59	0.99	0.81–1.23
Middle	1.27	0.90–1.79	0.99	0.79–1.23
Richer	1.32	0.96–1.82	0.91	0.74–1.14
Richest	1.50*	1.06–2.12	0.81	0.63– 1.04
Religious affiliation				
Other religion	1.00	–	1.00	–
CCAP	1.32	0.99–1.76	1.20	0.99–1.46
Catholic	1.31	0.99–1.73	1.21*	1.01–1.45
SDA/Baptist	1.26	0.83–1.92	1.25	0.95–1.64
Anglican	0.70	0.42–1.18	0.69	0.47–1.02
Muslim	0.72	0.47–1.09	1.90*	1.41–2.55
Number of living children				
Mean (S.D)	1.13*	1.04–1.23	0.91*	0.86–0.96
Partner age in years				
15–24	1.00	–	1.00	–
25–39	2.53	0.70–9.16	0.92	0.73–1.16
40–49	8.56*	2.31–31.76	1.19	0.89–1.60
50+	12.14*	3.14–46.95	0.94	0.61–1.45
Partner education				
No education	1.00	–	1.00	–
Primary	1.19	0.86–1.65	1.18	0.94–1.49
Secondary	0.74	0.51–1.08	0.94	0.71–1.23
High	0.73	0.39–1.38	0.55*	0.36–0.86

* *p* < 0.05; RRR, Relative Risk Ratio.

The results of the unadjusted model (Table 2) show that all the socio-demographic characteristics significantly predicted the use of female sterilisation, except the religious affiliation of women and the education of their partners. The age of the respondents significantly predicted the use of female sterilisation and SARCs, relative to LARCs. Being in the older ages (40 years and above) increased the relative likelihood of using female sterilisation by more than three times (RRR = 3.54, 95% CI = 2.61–4.80). However, adolescents and young women aged 15–24 years had a significantly lower likelihood of undergoing female sterilisation (RRR = 0.04, 95% CI = 0.01–0.11). The results also showed that women aged 40 years and above had an increased likelihood of using SARCs relative to LARCs when compared to women in the middle ages (25–39 years). With regards to educational attainment, women with secondary or higher education had a significantly lower likelihood of undergoing female sterilisation compared to those with no education (RRR = 0.45, 95% CI = 0.30–0.66). Furthermore, living in an urban area increased the relative likelihood of using female sterilisation by 47%. With respect to region of residence, women in the Southern (RRR = 0.55, 95% CI = 0.43–0.70) and Northern (RRR = 0.43, 95% CI = 0.30–0.61) regions had a relatively lower likelihood of using female sterilisation compared to those in the Central region.

Women in households in the richest wealth quintile had a significantly higher likelihood of using female sterilisation (RRR = 1.54, 95% CI = 1.10–2.16). In terms of religious affiliation, being Catholic increased the relative likelihood of undergoing female sterilisation by 32%. The results also showed that Catholic and Muslim women had an increased likelihood of using SARCs relative to LARCs. An increase in the mean number of children a woman had was associated with a greater likelihood of using female sterilisation. However, the average number of children a woman had was negatively associated with the use of SARCs. With respect to the characteristics of their partners, women with partners aged 40–49 years and those with partners 50 years and above had an increased likelihood of using female sterilisation compared to women with partners aged 15–24 years. The educational attainment of a woman's sexual partner did not predict the use of female sterilisation. However, women with partners of higher educational attainment had a significantly lower likelihood of using SARCs relative to LARCs.

The results of the adjusted model showed that while contraceptive autonomy increased the relative likelihood of using female sterilisation, health care autonomy was associated with a lower likelihood of using female sterilisation relative to LARCs. Health care autonomy did not predict the use of SARCs. Specifically, contraceptive decision-making autonomy increased the relative likelihood of using female sterilisation by 40% (RRR = 1.40, 95% CI = 1.05–1.86) and SARCs by 26% compared to using LARCs. Health care autonomy, on the other hand, reduced the relative likelihood of using female sterilisation by 40% (RRR = 0.60, 95% CI = 0.45–0.80). The results of the adjusted model also showed that all the socio-demographic characteristics that significantly predicted the use of female sterilisation in the unadjusted model maintained their significance as in the adjusted model as well the direction of their effect. The only exception was religious affiliation, where the observed difference between Catholics and Other Christian women disappeared.

## Discussion

This study examined the relationship between health care and contraceptive decision-making autonomy and the use of female sterilisation among married women in Malawi. Even though there are few studies in SSA on the relationship between women's decision-making autonomy and use of long-acting contraceptives, no study to date has examined whether health care and contraceptive decision-making autonomy predict the use of female sterilisation differently. By demonstrating that health care and contraceptive autonomy have different effects on the use of female sterilisation, this study provides useful insights and contributes to the literature on women's empowerment and contraceptive use.

The results of this study showed that a significantly low proportion of married women in Malawi had health care and contraceptive decision-making autonomy. Specifically, the study found that only about 18% and 13% of women had autonomy to make decisions on health care and contraceptive use respectively. For an overwhelming majority the women (80.6%) in this study, decisions to use contraceptives were made jointly with partners. While the finding that majority of contraceptive decisions were made jointly may suggest egalitarian decision making among couples, there is evidence that men still hold considerable sway in contraceptive decision-making in the sub-region including in Malawi (31–33). Even though the percentage (18.1%) of women that made health care decisions alone in this study was quite low, it was still higher than that reported for women in Senegal (6.3%) (25). The level of contraceptive autonomy in this study is slightly lower than that observed among women in high fertility regions of Ethiopia (34). It is important to understand that Malawi is one of the poorest countries in the world, with approximately 52% of the total population living below the national poverty line (35). The incidence of poverty is disproportionately high among women. The socio- economic disadvantages faced by Malawian women coupled with cultural and religious norms that vest decision-making power in men probably account for the low level of health care and contraceptive autonomy observed in this study.

The main finding of this study was that women who made contraceptive decisions alone had an increased likelihood of using female sterilisation while those who made health care decisions alone were less likely to be sterilized. This finding supports the hypothesis that women with autonomy in the contraceptive use domain have greater power in taking up female sterilisation than women with autonomy in general health care. The finding of this study is contrary to the study of Anita et al., in Uganda which found that women who reported their husband/partner as the main contraceptive decision maker were more likely to use female sterilisation compared to women that made contraceptive decisions alone (21). A plausible explanation of the finding of the current study is that women with greater autonomy in contraceptive decision making in Malawi are able to overcome the patriarchal norms that constraint women's use of female sterilisation in the sub-region. Nevertheless, women's autonomy to make decisions about health care did not translate into a greater likelihood of using female sterilisation. There is considerable evidence that norms and cultural practices associated with marriage or sexual partnership in many African societies constraint women's autonomy to determine the number of children to have, but not their autonomy pertaining to other domains such as engaging in economic activities or accessing health care services (36–38). Thus, the women with autonomy in health care in this study are probably limited when it comes to decision-making about using female sterilisation to effectively stop childbearing.

As expected, the results showed that younger women aged 15–24 years were less likely to undergo sterilisation while older women were significantly more likely to be sterilized relative to using LARCs. This finding is consistent with the results of previous studies conducted elsewhere (21, 39, 40). In a study among veterans in the USA, Arora et al., found that older women were more likely to take up sterilisation than use LARCs or short-term methods (39). In addition to the age of women, the current study also found that women with husbands/partners aged 40 years and above were significantly more likelihood to be sterilized relative to using LARCs. The increased likelihood of female sterilisation among older women and women with older partners in Malawi can be explained by the fact that older women or couples are more likely to have achieved their desired family size by these ages. Thus, such women and couples tend to be more likely to use female sterilisation to prevent any further childbearing.

Regarding education, the study found that women with secondary or higher education in Malawi were significantly less likely to be sterilized compared to women with no education. This finding is consistent with the study of Hayford et al., in the USA (41). It is known that increased educational attainment among women delays the onset of childbearing (42, 43), and this shift in the timing of childbearing can result in a lower demand for female sterilisation among such women. It is also possible that women with secondary or higher education are less likely to use female sterilisation because they can rely on themselves or their partners to use other effective contraceptive methods including LARCs and even vasectomy (41). With respect to place of residence, women in urban Malawi were significantly more likely to sterilized compared to their peers in rural areas. This finding was expected as urban areas tend to have more health facilities and the skilled professionals required to undertake female sterilisation. Similarly, women in the Northern and Southern regions were significantly less likely to be sterilized compared to those in the Central region. This result can also be explained by the low concentration of health facilities offering FP services in the northern and southern regions relative to the central region. For instance, only 14% of private health facilities with nurse midwives are in the northern and southern regions compared to 71% in the central region (44).

Furthermore, the study revealed that women in the richest wealth quintile had an increased likelihood of being sterilized compared to those in the poorest households. The women in the richest households in Malawi probably had the resources (including cost of transportation, and related fees) required to take up female sterilisation relative to those in poorest households. In addition to wealth, the current study found a positive association between the total number of children (parity) a woman had and the use of sterilisation. This finding was expected, and is consistent with the results of previous studies (21, 38, 45). Women with high order parity are more likely to have achieved their desired fertility, and therefore more likely to take up an effective and irreversible contraceptive such as female sterilisation to prevent further childbearing.

## Conclusion

The finding of this study demonstrates that health care and contraceptive decision making have different effects on the use of female sterilisation in Malawi. While women with autonomy in health care decision making had lower odds of using female sterilisation, those with contraceptive decision- making autonomy had higher odds of using female sterilisation. This finding has implications for research and women's empowerment programmes in settings such as Malawi. Firstly, research on autonomy and contraceptive use need to recognise the multi-dimensional nature of decision-making autonomy and the fact these dimensions could influence contraceptive use and method choice differently. Studies should therefore strive to include measures that capture various dimensions of autonomy in order to assess the pattern of their influence on specific contraceptive use behaviours. On the programmatic implications, the design and implementation of interventions aimed at improving women's decision-making autonomy need to identify and measure the specific outcomes that can be influenced by different aspects of the programme. Programmes that aim to increase autonomy in healthcare decision-making as a pathway to increasing the uptake of female sterilisation contraceptives may not achieve the desired impact.

## Study limitations

The main limitation of this study was the use of cross-sectional data which did not allow for the exploration of the causal pathway through which health care and contraceptive decision-making autonomy interacted with the use of female sterilisation. For instance, even though the results suggest that women with autonomy in contraceptive decision-making autonomy may be more likely to subsequently take up female sterilisation, it is also entirely possible that the use of female sterilisation increases contraceptive autonomy. Notwithstanding this limitation, this study may be one of the first to investigate whether healthcare and contraceptive decision-making autonomy predict the use of female sterilisation differently. The findings have provided insights on how these two dimensions of decision-making autonomy are differently associated with the uptake of female sterilisation relative to LARCs.

## Data Availability

Publicly available datasets were analyzed in this study. This data can be found here: https://dhsprogram.com/data/.
